# Assessing the efficacy of ultrasound-guided platelet-rich plasma on nerve regeneration and functional outcomes in forearm peripheral nerve injuries: a retrospective observational study

**DOI:** 10.3389/fneur.2026.1787289

**Published:** 2026-07-06

**Authors:** Qing Li, Cong Zou, Wenjie Liu, Lingling Zuo, Hui Long, Wei Gao, Bin Liu

**Affiliations:** 1 Department of Pain Treatment, The Second Affiliated Hospital of University of South China, Hengyang, Hunan, China; 2 Department of Anesthesiology, The Second Affiliated Hospital of University of South China, Hengyang, Hunan, China; 3 Department of Blood Transfusion, The Second Affiliated Hospital of University of South China, Hengyang, Hunan, China; 4 Department of Rehabilitation, The Second Affiliated Hospital of University of South China, Hengyang, Hunan, China

**Keywords:** disability evaluation, median nerve, peripheral nerve injuries, platelet-rich plasma, ulnar nerve, ultrasonography

## Abstract

**Background:**

This study evaluates the efficacy of ultrasound-guided platelet-rich plasma (PRP) treatment in promoting nerve regeneration and improving functional outcomes in patients with peripheral nerve injuries (PNI) of the forearm.

**Methods:**

A retrospective analysis was conducted on 183 patients diagnosed with unilateral forearm median or ulnar nerve injuries, classified as grade II or III according to the Sunderland system. Patients were divided into a conventional rehabilitation group (*n* = 105) and a PRP group (*n* = 78), which received additional ultrasound-guided PRP injections. Primary outcomes included nerve cross-sectional diameter, cross-sectional area, and Disabilities of the Arm, Shoulder, and Hand (DASH) scores. Secondary outcomes encompassed neurophysiological activity, motor and sensory function, and serum indicators of nerve function.

**Results:**

Post-treatment, the PRP group demonstrated a significantly larger nerve cross-sectional diameter (*p* = 0.021) and a significantly larger cross-sectional area (*p* = 0.004) compared to the conventional group. DASH scores were significantly lower in the PRP group at 2 months post-treatment (*p* = 0.008), indicating improved functional outcomes. Additionally, the PRP group exhibited higher sensory conduction velocity (*p* = 0.042) and motor conduction velocity (*p* = 0.044), along with improved Medical Research Council (MRC) muscle strength and British Medical Research Council (BMRC) sensory function scores at multiple follow-up points (all *p* < 0.05). Serum levels of brain-derived neurotrophic factor (BDNF) (*p* = 0.006), nerve growth factor (NGF) (*p* = 0.002), and neurotrophin-3 (NT-3) (*p* = 0.007) were also significantly elevated in the PRP group.

**Conclusion:**

Ultrasound-guided PRP treatment significantly enhances nerve regeneration and functional recovery in patients with forearm PNI compared to conventional rehabilitation alone, suggesting its potential as an effective adjunctive therapy.

## 1 Introduction

Peripheral nerve injuries represent a significant clinical challenge due to their potential for long-term disability and functional impairment (1). These injuries often result from trauma or surgical interventions and can severely affect an individual’s quality of life. The complexity of peripheral nerve regeneration poses considerable difficulties in achieving optimal recovery (2). Current therapeutic approaches mainly focus on surgical repair followed by rehabilitation (3). However, outcomes remain suboptimal with many patients experiencing incomplete recovery. This underscores the need for innovative adjunctive therapies that can enhance the healing process and improve functional outcomes (4).

The role of growth factors in tissue repair has been well documented in various medical fields. Platelet-rich plasma (PRP) contains high concentrations of these growth factors which are known to promote angiogenesis reduce inflammation and stimulate cellular proliferation (5). PRP has shown promise in enhancing wound healing and tissue regeneration in several musculoskeletal conditions such as tendon injuries and osteoarthritis (6, 7). Given its regenerative properties, PRP represents a candidate adjunctive therapy for treating peripheral nerve injuries (8). Previous studies have explored the use of PRP in nerve repair but evidence remains limited particularly regarding its application under ultrasound guidance which could ensure precise delivery to the injury site (9, 10).

Ultrasound technology provides real-time imaging allowing for accurate localization of the target area during PRP injection. This precision is crucial for peripheral nerve injuries where exact placement of therapeutic agents can significantly influence treatment efficacy. Ultrasound-guided injections have been utilized in various clinical settings including pain management and musculoskeletal disorders demonstrating improved outcomes (11, 12). By leveraging this technology, PRP administration could be optimized leading to enhanced nerve regeneration and functional recovery (13). Despite these advancements the integration of PRP with ultrasound guidance for peripheral nerve injuries remains underexplored highlighting the necessity for further investigation into this promising approach.

Moreover, understanding the impact of PRP on both structural and functional aspects of nerve recovery is essential. Changes in nerve cross-sectional diameter and area serve as key indicators of nerve regeneration (14). These metrics provide insight into the extent of tissue repair and remodeling following injury. In addition, patient-reported outcomes such as the Disabilities of the Arm, Shoulder, and Hand (DASH) score offer valuable perspectives on functional recovery and quality of life (15). Evaluating these parameters can help elucidate the comprehensive benefits of PRP treatment beyond mere anatomical improvements. Assessing both objective and subjective measures allows for a more holistic understanding of treatment efficacy and patient satisfaction. Therefore, investigating the effects of ultrasound-guided PRP on peripheral nerve injuries through these multiple dimensions is critical to advancing therapeutic strategies in this field.

While PRP has shown promise in treating upper extremity neuropathies for more than a decade, there is limited research on its application in other contexts, such as forearm nerve injuries (9, 16). In addition, variability in study designs and outcomes raises questions about the generalizability of findings, emphasizing the need for more standardized research in this area (17). Despite the theoretical benefits of ultrasound-guided PRP, there is limited evidence supporting its effectiveness in promoting nerve regeneration and functional recovery in patients with forearm PNI (18). Understanding these mechanisms and their clinical implications will support the development of more effective treatments.

This study aims to fill this gap by evaluating the impact of this treatment modality. The primary objective is to assess the efficacy of ultrasound-guided PRP in promoting nerve regeneration and improving functional outcomes in patients with grade II and III median or ulnar nerve injuries in the forearm. By investigating these parameters, this research aims to contribute valuable insights into the potential of PRP as a standard adjunctive therapy for peripheral nerve injuries, ultimately improving patient care and outcomes.

## 2 Materials and methods

### 2.1 Study population

**Figure 1:**
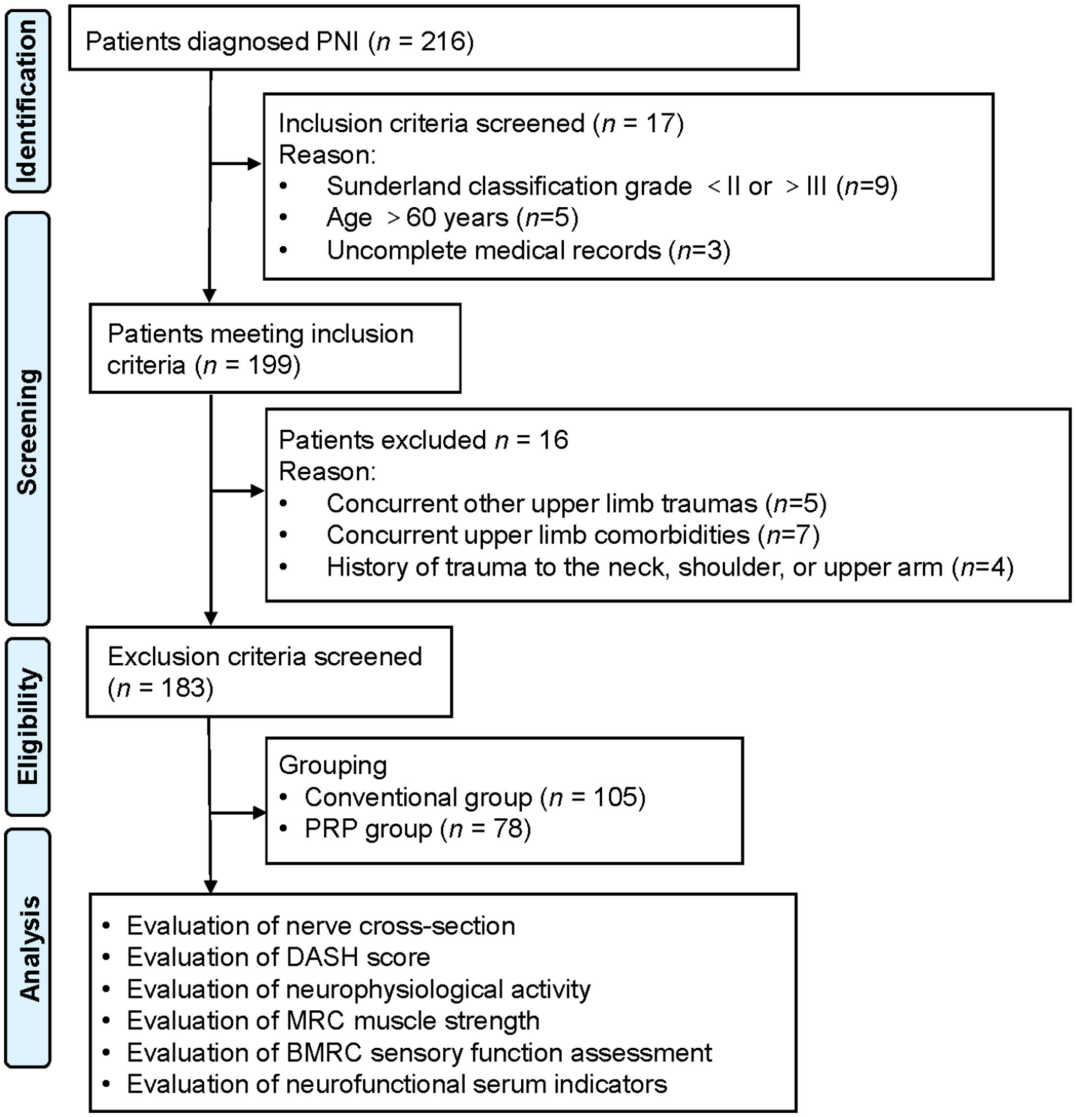
Flowchart.

A retrospective analysis was conducted on 183 patients with peripheral nerve injuries (PNI) admitted to our hospital from January 10, 2020 to January 10, 2025 (Figure 1). Inclusion criteria were: ① met the diagnostic criteria for PNI (19), and diagnosed with unilateral forearm median or ulnar nerve injury; ② confirmed as grade II or III injury according to the Sunderland classification system through clinical and electrophysiological evaluation; ③ had received nerve repair surgery; ④ aged between 18 and 60 years; ⑤ complete medical records without any missing information. Exclusion criteria included: ① concurrent other upper limb traumas (burns, crush injuries, etc.); ② poor skin condition at the injection site (unhealed surgical wounds, skin ulcers, or skin defects at the nerve injury site); ③ concurrent fractures or major vascular injuries; ④ concurrent upper limb comorbidities (such as cervical spondylosis, soft tissue injuries, or masses, etc.); ⑤ history of trauma to the neck, shoulder, or upper arm; ⑥ previous history of peripheral nerve graft surgery; ⑦ pregnant or breastfeeding women; ⑧ history of systemic diseases (such as diabetes, patients undergoing chemotherapy for tumors, degenerative joint disease, demyelinating diseases of the peripheral nerves, etc.) or insufficient function of heart, liver, kidney, or lung; ⑨ cognitive or mental disorders.

### 2.2 Study grouping

All patients began a 12-week adjunctive treatment regimen starting from 8 weeks post-surgery. Depending on the type of adjunctive treatment received after surgery, the 183 patients were divided into a conventional group (*n* = 105) and a platelet-rich plasma (PRP) group (*n* = 78). The conventional group was defined as patients who only received standard rehabilitation treatment, whereas the PRP group was defined as patients who, in addition to standard rehabilitation treatment, also received ultrasound-guided PRP injections. This study was conducted in accordance with the Declaration of Helsinki and was reviewed and approved by the Ethics Committee of the Second Affiliated Hospital of University of South China (Approval number: 2025037). Given the retrospective nature of the study (using existing de-identified clinical data), the Ethics Committee waived the requirement for individual informed consent in accordance with the Guidelines for the Protection of Human Subjects in Medical Research.

### 2.3 Treatment

*Conventional rehabilitation treatment included*: ① Low-intensity ultrasound therapy was administered using the Japanese Ito US-751 Ultrasound Therapy Device (Shanghai Baijun Health Technology Co., Ltd.). During treatment, an output power of 30 mW/cm^2^, a frequency of 1.5 MHz, and a pulse width of 200 μs were selected. Each session lasted for 15 min, once daily, 5 days per week; ② Low-frequency electrical stimulation was applied using the RH-JPSJ-B Transcutaneous Electrical Nerve Stimulation Device (Shanghai Huanxi Medical Equipment Co., Ltd.). Electrodes were sequentially placed on the proximal and distal skin areas along the injured nerve pathway. The current intensity was set between 30 to 40 mA, adjusted according to patient tolerance. Each session lasted for 20 min, once daily, 5 days per week; ③ Patients were guided to actively engage in early functional exercises, including joint flexion and extension, full range of motion exercises, and skin sensation training. Each session lasted from 40 to 60 min, once daily, 5 days per week; ④ Oral mecobalamin tablets (Approval No. H20051440, Jiangxi Kerui Pharmaceutical Co., Ltd., Jiangxi Province) were administered at a dosage of 0.5 mg per time, three times daily, for a continuous treatment period of 12 weeks.

*Ultrasound-guided PRP treatment*: Prior to treatment, patients were confirmed to have no coagulation abnormalities or infectious diseases. A total of 8 mL of autologous venous blood was collected from the antecubital vein and placed in a centrifuge (SF-TDL4A-5C, Arthrex Inc., USA). The process involved two stages: In the first stage, the blood was centrifuged at 2,000 rpm for 5 min to separate red blood cells from the plasma-platelet layer. The upper plasma-platelet mixture was then transferred to a new sterile centrifuge tube. In the second stage, the mixture was centrifuged at 5,000 rpm for 10 min, and the upper 2/3 of platelet-poor plasma (PPP) was discarded, leaving the lower 1/3 rich in platelets, which constituted the PRP (4 mL). The resulting PRP was leukocyte-poor (LP-PRP), as the double-centrifugation protocol with discarding of the upper 2/3 of platelet-poor plasma effectively removes most leukocytes. The platelet concentration in the PRP was measured for every patient using a hematology analyzer (XN-1000, Sysmex Corp., Japan) to ensure it reached 3–5 times the baseline value (typically 1.0 × 10^11^–1.5 × 10^11^/L) before use for injection. Under ultrasound guidance (Resona 7, Mindray Bio-Medical Electronics Co., Ltd., China) using a 4–15 MHz high-frequency linear probe (L15-4S) with imaging parameters optimized for peripheral nerve visualization (frequency: 12 MHz; gain: 60–70 dB; dynamic range: 65 dB), a 22G sterile needle (305157, BD Inc., USA) was inserted using an in-plane approach, with the needle trajectory aligned parallel to the long axis of the ultrasound probe. The needle tip was advanced under real-time visualization until it reached the epifascicular epineurium—the outermost hyperechoic connective tissue layer surrounding the nerve. The injection was performed perineurally (extraneurally), i.e., outside the epineurium, without penetrating the nerve parenchyma. The target was the loose connective tissue space containing the vasa nervorum (the nerve’s microvascular network) (20). Needle position was confirmed by gentle hydrodissection with 0.5 mL of normal saline, which produced a hypoechoic halo separating the nerve from surrounding tissues, confirming that the needle tip was extraneural. After confirmation, the activated PRP (injectable fibrin clot) was slowly injected while monitoring the spread of the injectate as a hyperechoic halo around the nerve, without intraneural infiltration. This perineural approach was chosen to maximize safety, target the vasa nervorum, and achieve mechanical neurolysis via hydrodissection.

### 2.4 Evaluation metrics

**Figure 2:**
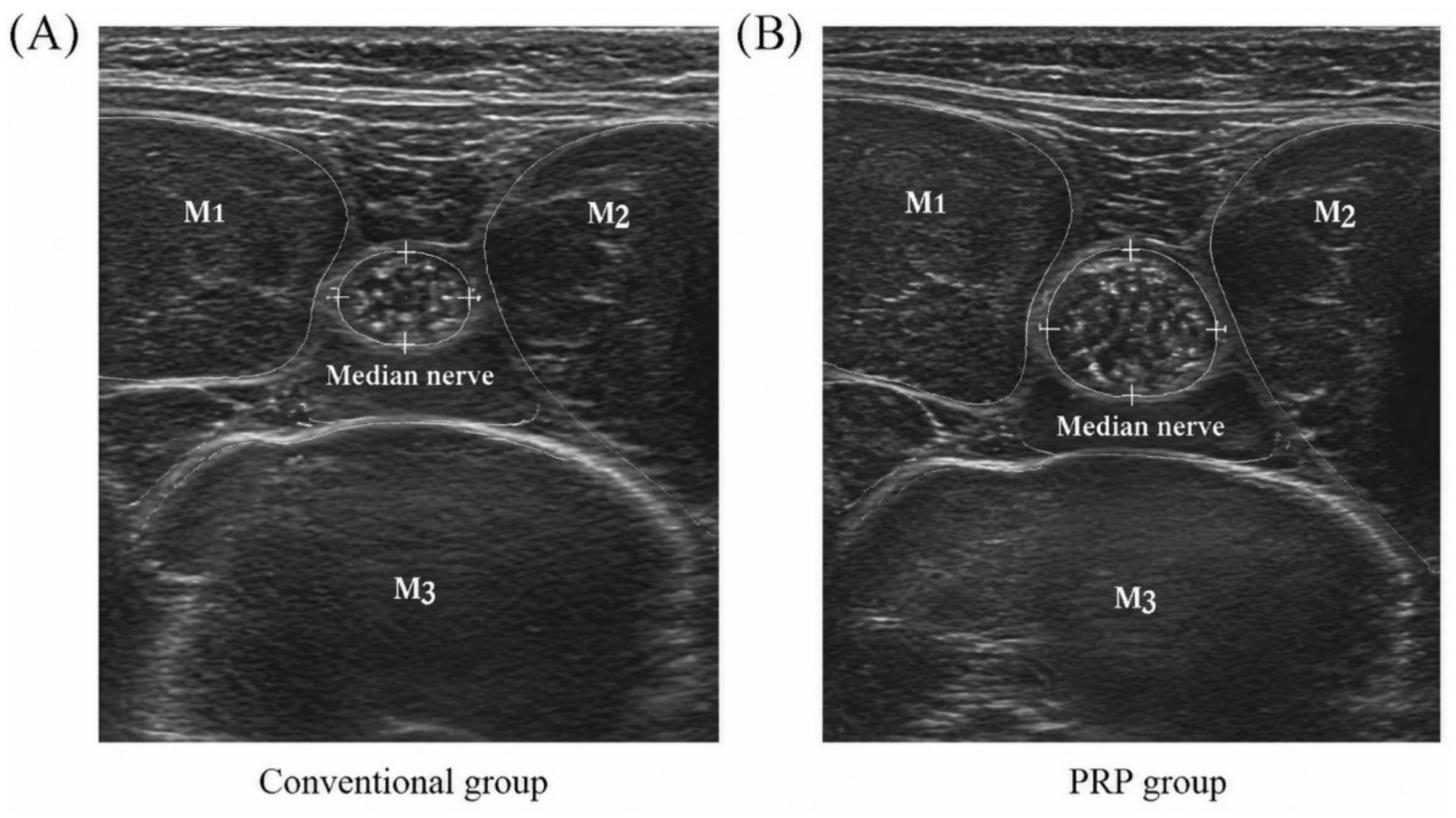
Ultrasound measurement of nerve cross-sectional diameter and area. **(A)** Representative sonographic image from the conventional group showing the median nerve at the injury site; **(B)** representative sonographic image from the PRP group showing the median nerve at the injury site. PRP, platelet-rich plasma.

The primary evaluation metrics of this study were the nerve cross-sectional diameter and area, as well as the Disabilities of the Arm, Shoulder, and Hand (DASH) score, including measurements taken at pre-treatment and post-12 weeks of treatment. Ultrasound (same as in Section 2.3) was used to measure the nerve cross-sectional diameter and area (Figure 2). The nerve cross-sectional diameter was measured in the transverse view as the maximum transverse diameter (i.e., the longest distance between the two outer borders of the hyperechoic epineurial rim perpendicular to the nerve’s long axis), using the system’s electronic caliper. The cross-sectional area was measured in the transverse view by manually tracing the inner edge of the hyperechoic epineurial rim (i.e., the boundary between the epineurium and the perineurium/fascicles) using the system’s electronic caliper and free-hand tracing tool. This approach excludes the outer epineurium to minimize measurement variability. All ultrasound measurements (nerve cross-sectional diameter and area) were performed by the same experienced musculoskeletal radiologist who was blinded to group allocation. The DASH score was employed to assess functional disability, symptom severity, and the impact on daily life following upper limb injury. The DASH questionnaire consists of 30 items, each rated on a 6-point Likert scale where 1 represents no difficulty/no symptoms and 6 represents inability to perform/unbearable symptoms. The scores for all completed items are summed, averaged, and then converted to a standardized score ranging from 0 to 100 using the formula: DASH score = [(average score - 1) ÷ 5] × 100. Lower scores indicate better upper limb function and higher quality of life. The Cronbach’s alpha coefficient for this scale is 0.94 (21).

Secondary evaluation metrics included neurophysiological activity, motor function, sensory function, and serum indicators of nerve function. Before and after treatment, a four-channel electromyography evoked potential instrument (M8000C, Shandong Oulibo Medical Equipment Co., Ltd., China) was used to assess the patients’ neurophysiological activity, including sensory conduction velocity (SCV) and motor conduction velocity (MCV). At pre-treatment, post-4 weeks of treatment, post-8 weeks of treatment, and post 12 weeks of treatment, the Medical Research Council (MRC) muscle strength grading criteria and the British Medical Research Council (BMRC) sensory function assessment criteria were employed to evaluate the recovery of motor and sensory functions. The MRC muscle strength grading ranges from 0 to 5, corresponding to scores of 0 to 5, with higher scores indicating better motor function. The BMRC sensory function assessment includes six levels: S0, S1, S2, S3, S3+, and S4, corresponding to scores of 0 to 5, with higher scores indicating better sensory function. At pre-treatment and post-12 weeks of treatment, a multifunctional microplate reader (Thermo Varioskan LUX, Thermo Fisher Scientific Inc., USA) was used to measure serum indicators of nerve function. The samples analyzed were 2 mL of anticoagulated and centrifuged serum, using enzyme-linked immunosorbent assay (ELISA) methods. The measured indicators included brain-derived neurotrophic factor (BDNF), nerve growth factor (NGF), and neurotrophin-3 (NT-3). Supplementary Video 1 visualizes the ultrasound-guided procedures and the biological effects of PRP on the injured peripheral nerve.

### 2.5 Statistical analysis

This study used SPSS statistical software (Version 29.0; developed by SPSS Inc., Chicago, IL, USA) for statistical analysis. After validation with the Shapiro–Wilk test, continuous variables in this study were found to follow a normal distribution and are presented as mean ± standard deviation (M ± SD). Independent samples t-tests were used for comparisons between groups. Categorical variables are presented as frequencies and percentages [*n* (%)] and were compared between groups using the *χ*^2^ test. A *p-*value less than 0.05 was considered statistically significant.

Given the retrospective nature of this study, a formal prospective sample size calculation was not performed. The sample size was determined by including all consecutive patients who met the inclusion and exclusion criteria during the specified study period (January 10, 2020 to January 10, 2025). A *post-hoc* power analysis was conducted using G*Power software (version 3.1.9.7) based on the observed effect size in the primary outcome (nerve cross-sectional area change) between groups. With an effect size of 0.45, an alpha level of 0.05, and the achieved sample sizes (conventional group: *n* = 105, PRP group: *n* = 78), the achieved statistical power was calculated to be >0.95, indicating that the study had sufficient power to detect the observed differences.

## 3 Results

### 3.1 General information

The comparison of general information between the conventional group and the PRP group (Table 1) showed no significant differences in any of the assessed parameters, including age, gender, body mass index (BMI), educational level, manual labor intensity, smoking history, drinking history, injury side, injured nerve, injury degree, and injury to surgery time (all *p* > 0.05). These results indicated that the baseline characteristics were comparable between the two groups, supporting the comparability of the study populations at the start of the intervention.

**Table 1:** Comparison of general information between two groups.

Parameters	Conventional group (*n* = 105)	PRP group (*n* = 78)	*t*/*χ*^2^	*p*
Age (years)	41.36 ± 8.57	40.89 ± 9.24	0.352	0.725
Gender [*n* (%)]			0.072	0.789
Male	68 (64.76%)	52 (66.67%)		
Female	37 (35.24%)	26 (33.33%)		
BMI (kg/m^2^)	24.15 ± 2.02	23.87 ± 1.91	0.960	0.339
Educational level [*n* (%)]			0.348	0.840
Junior high school or below	38 (36.19%)	25 (32.05%)		
Senior high school or secondary vocational school	45 (42.86%)	36 (46.15%)		
Junior college or above	22 (20.95%)	17 (21.79%)		
Manual labor [*n* (%)]			0.698	0.705
Low	40 (38.10%)	32 (41.03%)		
Moderate	35 (33.33%)	28 (35.90%)		
High	30 (28.57%)	18 (23.08%)		
Smoking history [*n* (%)]			0.267	0.605
Yes	43 (40.95%)	29 (37.18%)		
No	62 (59.05%)	49 (62.82%)		
Drinking history [*n* (%)]			0.203	0.652
Yes	37 (35.24%)	25 (32.05%)		
No	68 (64.76%)	53 (67.95%)		
Injury side [*n* (%)]			0.102	0.750
Left	50 (47.62%)	39 (50.00%)		
Right	55 (52.38%)	39 (50.00%)		
Injured nerve [*n* (%)]			0.129	0.720
Median nerve	58 (55.24%)	41 (52.56%)		
Ulnar nerve	47 (44.76%)	37 (47.44%)		
Injury degree [*n* (%)]			0.498	0.480
II	54 (51.43%)	36 (46.15%)		
III	51 (48.57%)	42 (53.85%)		
Injury to surgery time (days)	7.82 ± 2.15	8.11 ± 1.97	0.951	0.343

PRP, platelet-rich plasma; BMI, body mass index.

### 3.2 Nerve cross-section

**Figure 3:**
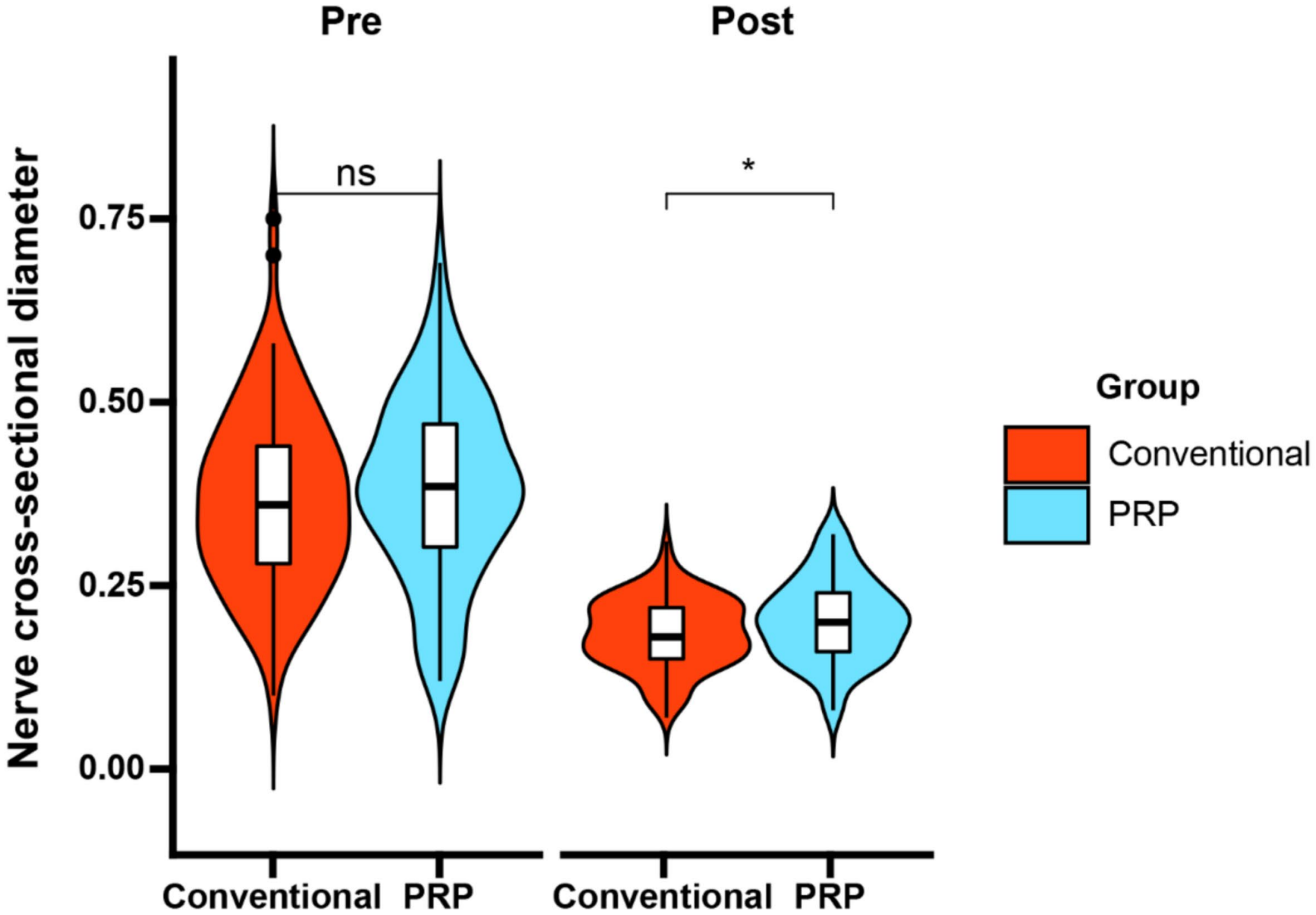
Comparison of nerve cross-sectional diameter between two groups (cm). ns, no significant difference; *p < 0.05. PRP, platelet-rich plasma.

The comparison of nerve cross-sectional diameter between the conventional group and the PRP group (Figure 3) showed no significant differences at baseline (*p* > 0.05). However, post-treatment, the PRP group exhibited a significantly larger diameter (0.20 ± 0.06 vs. 0.18 ± 0.05, *t* = 2.334, *p* = 0.021).

**Figure 4:**
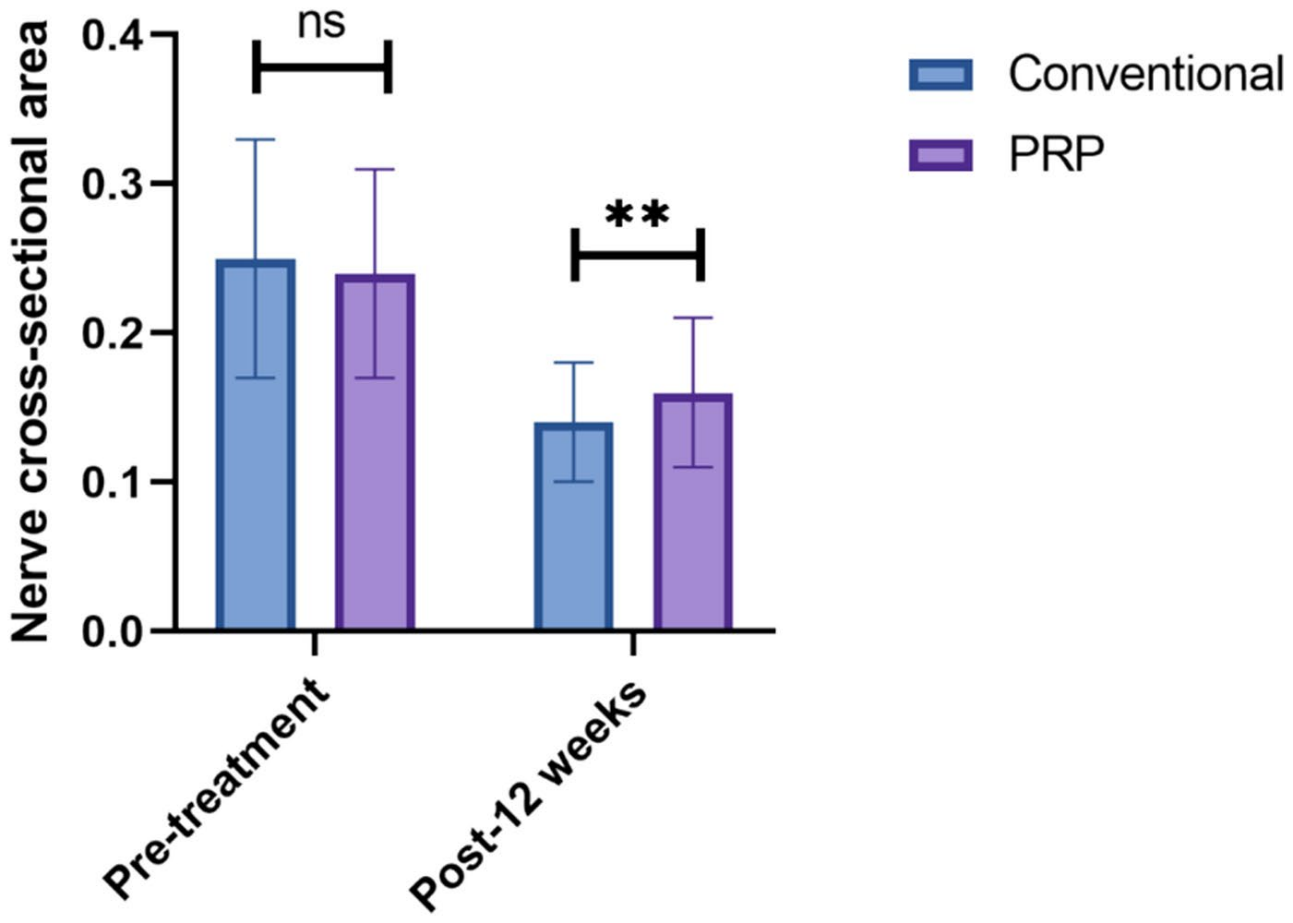
Comparison of nerve cross-sectional area between two groups (cm^2^). ns, no significant difference; **p < 0.01. PRP, platelet-rich plasma.

The comparison of nerve cross-sectional area between the conventional group and the PRP group (Figure 4) showed no significant differences at baseline (*p* > 0.05). However, post-treatment, the PRP group exhibited a significantly larger area (0.16 ± 0.05 vs. 0.14 ± 0.04, *t* = 2.920, *p* = 0.004).

### 3.3 DASH score

At pre-treatment, there was no significant difference in DASH scores between the two groups (*p* > 0.05) (Table 2). Two months after treatment, the PRP group showed significantly lower DASH scores (35.42 ± 5.73 vs. 37.97 ± 6.84, *t* = 2.678, *p* = 0.008), indicating better functional outcomes compared to the conventional group.

**Table 2:** Comparison of DASH score between two groups (scores).

Parameters	Conventional group (*n* = 105)	PRP group (*n* = 78)	*t*	*p*
Pre-treatment	85.83 ± 7.51	86.29 ± 6.92	0.420	0.675
Post-12 weeks of treatment	37.97 ± 6.84	35.42 ± 5.73	2.678	0.008

DASH, disabilities of the arm, shoulder and hand; PRP, platelet-rich plasma.

### 3.4 Neurophysiological activity

**Figure 5:**
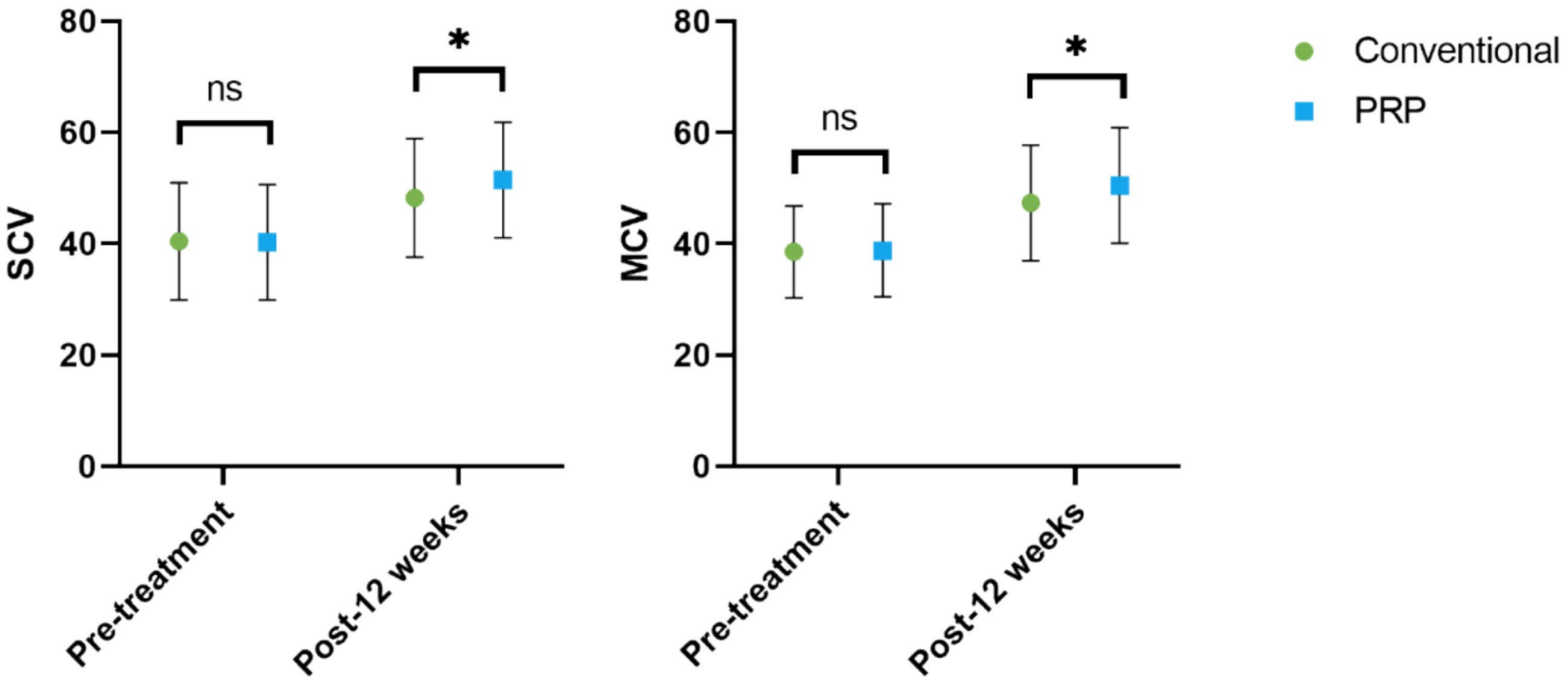
Comparison of neurophysiological activity between two groups (cm/s). ns, no significant difference; *p < 0.05. PRP, platelet-rich plasma; SCV, sensory conduction velocity; MCV, motor conduction velocity.

**Figure 6:**
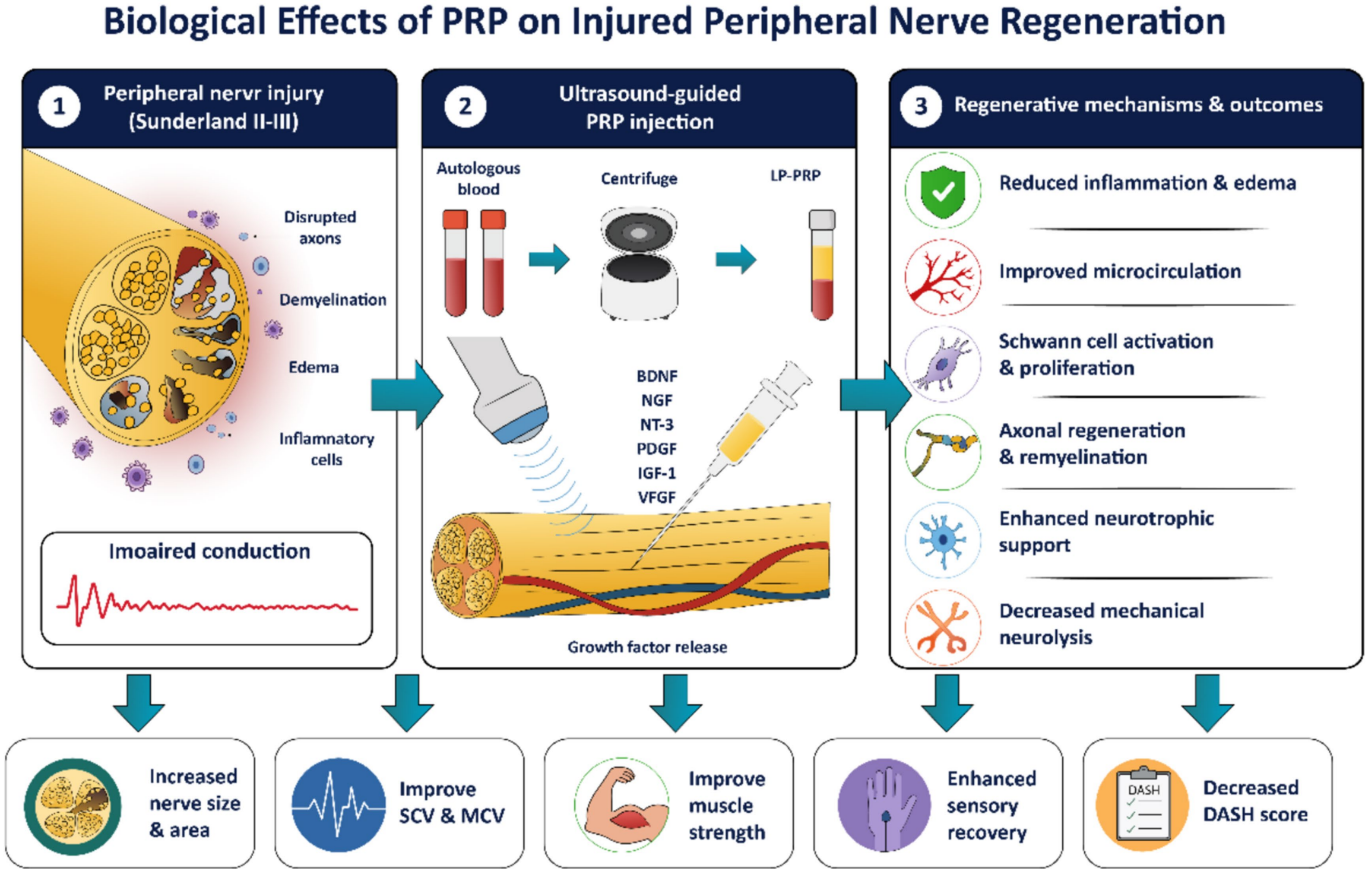
Schematic illustration of the biological effects of PRP on the injured peripheral nerve.

The comparison of neurophysiological activity between the conventional group and the PRP group (Figure 5) showed no significant differences at baseline for either SCV or MCV (both *p* > 0.05). Post-treatment, the PRP group exhibited significantly higher SCV (51.46 ± 10.38 vs. 48.23 ± 10.68, *t* = 2.049, *p* = 0.042) and MCV (50.52 ± 10.39 vs. 47.37 ± 10.41, *t* = 2.025, *p* = 0.044). Figure 6 schematically illustrates the biological effects of PRP on the injured peripheral nerve.

### 3.5 Neuromotor function

**Figure 7:**
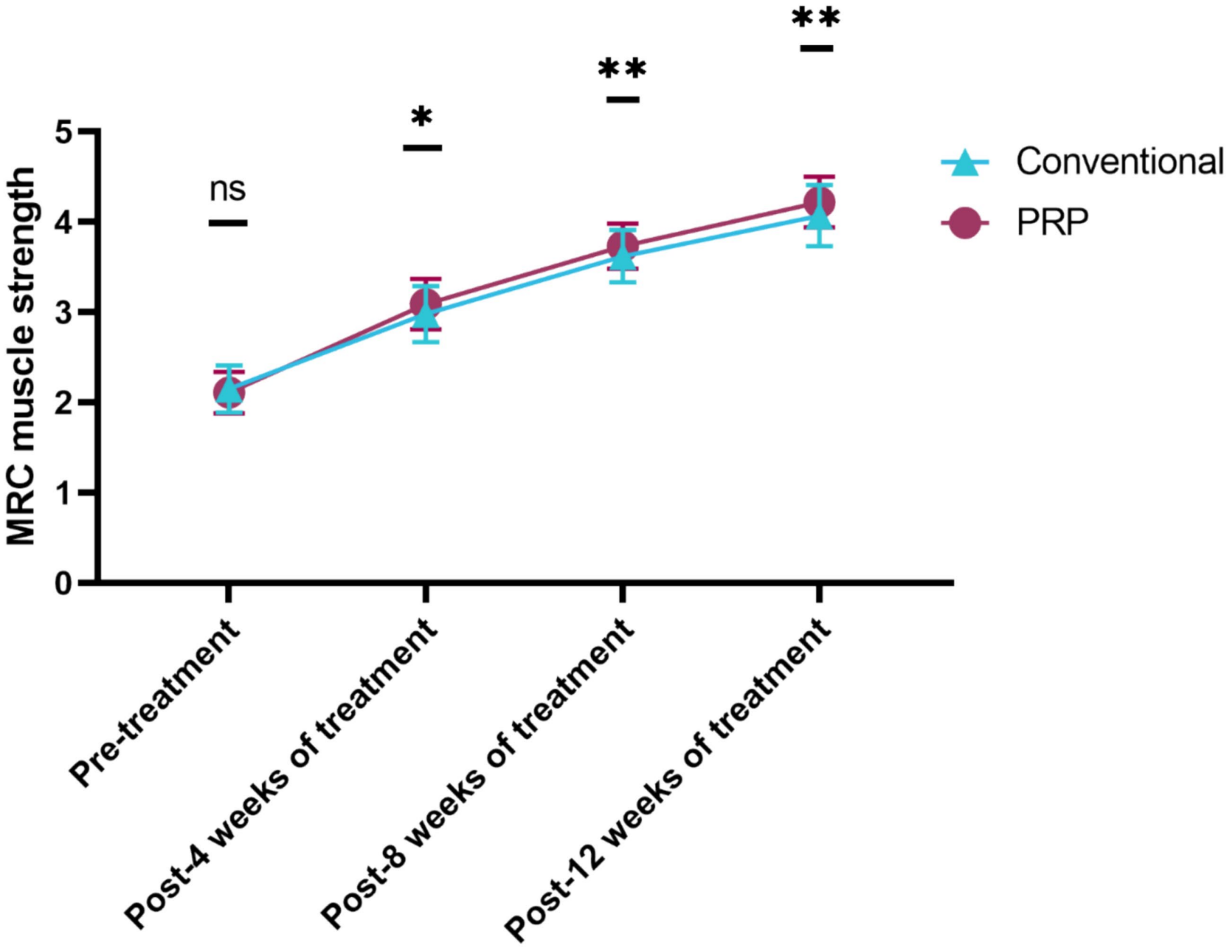
Comparison of MRC muscle strength between two groups (scores). ns, no significant difference; *p < 0.05; **p < 0.01. MRC, Medical Research Council; PRP, platelet-rich plasma.

At pre-treatment, there was no significant difference in MRC muscle strength between the two groups (*p* > 0.05) (Figure 7). However, 4 weeks after treatment, the PRP group demonstrated significantly higher MRC muscle strength (3.09 ± 0.28 vs. 2.98 ± 0.31, *t* = 2.372, *p* = 0.019). This trend continued with progressively greater differences observed at 8 weeks (3.73 ± 0.25 vs. 3.62 ± 0.29, *t* = 2.644, *p* = 0.009) and 12 weeks (4.22 ± 0.28 vs. 4.07 ± 0.34, *t* = 3.335, *p* = 0.001) post-treatment.

### 3.6 Neurosensory function

The comparison of BMRC sensory function assessment between the conventional group and the PRP group (Table 3) showed no significant differences at baseline (*p* > 0.05). Post-treatment, the PRP group exhibited significantly higher BMRC sensory function scores at 4 weeks (2.12 ± 0.25 vs. 2.01 ± 0.29, *t* = 2.788, *p* = 0.006), 8 weeks (3.56 ± 0.28 vs. 3.41 ± 0.43, *t* = 2.762, *p* = 0.006), and 12 weeks (4.03 ± 0.24 vs. 3.93 ± 0.16, *t* = 3.105, *p* = 0.002).

**Table 3:** Comparison of BMRC sensory function assessment between two groups (scores).

Parameters	Conventional group (*n* = 105)	PRP group (*n* = 78)	*t*	*p*
Pre-treatment	0.86 ± 0.26	0.88 ± 0.23	0.561	0.576
Post-4 weeks of treatment	2.01 ± 0.29	2.12 ± 0.25	2.788	0.006
Post-8 weeks of treatment	3.41 ± 0.43	3.56 ± 0.28	2.762	0.006
Post-12 weeks of treatment	3.93 ± 0.16	4.03 ± 0.24	3.105	0.002

BMRC, British Medical Research Council; PRP, platelet-rich plasma.

### 3.7 Neurofunctional serum indicators

At pre-treatment, there were no significant differences in BDNF (*p* > 0.05), NGF (*p* > 0.05), or NT-3 (*p* > 0.05) levels between the two groups (Table 4). After treatment, the PRP group showed significantly higher levels of BDNF (12.48 ± 3.52 vs. 11.17 ± 2.57, *t* = 2.773, *p* = 0.006), NGF (45.24 ± 10.35 vs. 40.28 ± 10.52, *t* = 3.176, *p* = 0.002), and NT-3 (173.56 ± 20.27 vs. 165.35 ± 20.23, *t* = 2.711, *p* = 0.007) compared to the conventional group.

**Table 4:** Comparison of neurofunctional serum indicators between two groups.

Parameters	Conventional group (*n* = 105)	PRP group (*n* = 78)	*t*	*p*
BDNF (μg/L)				
Pre-treatment	7.55 ± 1.73	7.74 ± 1.56	0.773	0.441
Post-12 weeks of treatment	11.17 ± 2.57	12.48 ± 3.52	2.773	0.006
NGF (pg/mL)				
Pre-treatment	30.52 ± 5.34	30.36 ± 5.27	0.204	0.839
Post-12 weeks of treatment	40.28 ± 10.52	45.24 ± 10.35	3.176	0.002
NT-3 (pg/mL)				
Pre-treatment	133.57 ± 10.36	132.41 ± 10.52	0.741	0.459
Post-12 weeks of treatment	165.35 ± 20.23	173.56 ± 20.27	2.711	0.007

PRP, platelet-rich plasma; BDNF, brain-derived neurotrophic factor; NGF, nerve growth factor; NT-3, neurotrophin-3.

## 4 Discussion

Peripheral nerve injuries represent a significant clinical challenge due to their potential for long-term disability and functional impairment. The current study evaluated the efficacy of ultrasound-guided PRP treatment in patients with forearm peripheral nerve injuries, focusing on structural, functional, and serum-level outcomes. Our findings indicate that adjunctive PRP treatment may enhance nerve regeneration and functional recovery compared to conventional rehabilitation alone. However, these results must be critically contextualized within the evolving landscape of PRP research, acknowledging both supportive and conflicting evidence, and considering the biological mechanisms and methodological variables that may influence outcomes.

The observed increases in both nerve cross-sectional diameter and cross-sectional area in the PRP group suggests improved nerve remodeling. While nerve edema typically increases diameter without proportionally increasing cross-sectional area, the simultaneous increases in both parameters in the PRP group suggest a fundamentally different biological process: axonal sprouting, Schwann cell proliferation, and remyelination (8, 22). PRP-derived growth factors such as (PDGF) and vascular endothelial growth factor (VEGF) promote Schwann cell proliferation and increase vascularization around the nerve, both of which contribute to increased cross-sectional dimensions on ultrasound (23, 24). The smaller diameter observed in the conventional group may represent a more atrophic nerve with less regenerative activity, whereas the PRP group’s larger diameter reflects active tissue rebuilding. These structural findings align with several recent animal and clinical studies reporting enhanced nerve regeneration with PRP (25, 26). However, it is important to note that some clinical trials, such as the randomized controlled trial by Chen et al. (16) on carpal tunnel syndrome, reported significant symptomatic improvement but no significant difference in nerve conduction studies compared to controls. This discrepancy highlights the variability in outcome measures and suggests that structural improvements may not always correlate linearly with electrophysiological or functional gains.

The improvement in DASH scores in the PRP group highlights potential functional benefits, consistent with studies reporting PRP’s efficacy in upper extremity conditions (27, 28). Nevertheless, the literature is not unanimous. A systematic review by Jerome & Matsui noted that while PRP shows promise, evidence remains heterogeneous due to differences in injury severity, PRP formulations, and outcome timing (17). Our study adds to this body of evidence but should be interpreted with caution given the retrospective design and lack of a sham control, which may introduce bias in patient-reported outcomes such as DASH.

The clinical interpretability of our findings is enhanced by anchoring the observed improvements to established minimal clinically important difference (MCID) thresholds where available. For the DASH score, our observed mean improvement in the PRP group (50.87 points) substantially exceeded the widely accepted MCID range of 10–14 points (29–31), indicating that the magnitude of functional recovery was clearly clinically meaningful. However, the additional between-group improvement with PRP over conventional rehabilitation alone (2.55 points) did not reach the MCID threshold, suggesting that the statistically significant difference observed (*p* = 0.008) may not translate into a clinically perceptible advantage for patients. This finding does not diminish the value of PRP as an adjunctive therapy—both groups achieved clinically meaningful improvements from baseline—but it does highlight that the incremental benefit of adding PRP to an already effective conventional rehabilitation protocol may be modest in magnitude.

The enhancements in sensory and motor conduction velocities (SCV, MCV) in our PRP group suggest improved myelination and axonal integrity. Similar findings were reported by Zhu et al. (18) using multimodal ultrasound and PRP in nerve crush models. However, other studies, including work by Rossi et al. (28) on tendinopathy, remind us that PRP’s effects may be condition- and protocol-dependent. Variability in PRP preparation (such as centrifugation speed, platelet concentration, activation method, and the use of leukocyte-rich vs. leukocyte-poor PRP) can significantly influence growth factor release and biological activity (5, 7). Our protocol used a double-spin method with calcium chloride activation, yielding a platelet concentration 3–5 times baseline, but differences in preparation across studies may contribute to inconsistent results in the literature.

At a mechanistic level, PRP is thought to facilitate nerve repair through multiple coordinated pathways. Growth factors such as BDNF, NGF, and NT-3 (significantly elevated in our PRP group) play major roles in neuronal survival, axonal guidance, and synaptic plasticity (32, 33). In particular, BDNF promotes Schwann cell migration and myelination, while NGF supports sensory neuron survival (34). PRP may also modulate the inflammatory microenvironment by reducing pro-inflammatory cytokines and enhancing angiogenesis, thereby creating a more conducive niche for regeneration (35, 36). Recent reviews, such as that by Shang et al. (8), discuss how PRP-derived exosomes and microvesicles may further contribute to intercellular communication and regenerative signaling. A deeper exploration of these cellular and molecular interactions, such as the role of PRP in macrophage polarization toward a pro-regenerative (M2) phenotype, would strengthen the mechanistic rationale for its use (37).

Our results suggest that adding ultrasound-guided perineural PRP injections to a standard 12-week rehabilitation program may offer modest but statistically significant benefits in terms of nerve structural remodeling (increased cross-sectional diameter and increased cross-sectional area), neurophysiological recovery (higher SCV and MCV), and early functional gains (lower DASH scores, improved MRC and BMRC grades) compared with rehabilitation alone. However, several clinical considerations must be highlighted. First, the between-group difference in DASH scores (2.55 points) did not reach the established MCID threshold of 10–14 points, indicating that the incremental benefit of PRP over conventional rehabilitation alone, while statistically significant, may not be perceptible to patients in terms of daily function. Second, both groups achieved substantial improvements from baseline—far exceeding MCID thresholds—underscoring that structured rehabilitation remains the cornerstone of functional recovery. Third, the perineural (extraneural) injection technique used in this study is safe, minimally invasive, and can be performed in an outpatient setting under ultrasound guidance, which may be particularly valuable for patients who have reached a plateau with conventional therapy or those who are not candidates for surgical revision. From a clinical decision-making perspective, PRP may be considered as an adjunctive option for motivated patients with persistent symptoms or delayed recovery after primary nerve repair, but it should not replace established rehabilitation protocols. Future studies are needed to identify which patient subgroups (e.g., those with greater initial injury severity, or those with poor early recovery) derive the most clinically meaningful benefit from PRP.

Performing accurate perineural injections requires mastering needle-probe alignment, real-time hand-eye coordination, and recognition of epineurial vs. fascicular anatomy. Common beginner pitfalls include loss of needle tip visibility and unintentional intraneural penetration. As highlighted by Salce et al. (38), structured simulation training using phantoms (e.g., gelatin-based or commercial models) significantly accelerates skill acquisition and improves safety. We recommend at least 10–15 supervised phantom trials before clinical application, followed by proctored patient procedures until consistent needle visualization and extraneural targeting are achieved.

This study has several limitations that should be acknowledged. Major limitations include: (1) the retrospective, non-randomized design, which introduces potential selection bias and limits the ability to establish causality; (2) the absence of a placebo (sham injection) control group, meaning that the observed benefits of PRP cannot be entirely separated from a possible placebo effect, particularly for patient-reported outcomes such as DASH scores; (3) the relatively modest sample size (*n* = 78 in the PRP group), which may have been underpowered to detect small but clinically relevant differences in secondary outcomes; (4) the lack of blinding of patients and outcome assessors, which could have introduced detection bias; and (5) the single-center design, which limits generalizability to other populations and healthcare settings. Minor limitations include: (1) the absence of long-term follow-up beyond 12 weeks, so the durability of PRP’s effects remains unknown; (2) the use of a single PRP preparation protocol (double-spin, calcium chloride activation, 3–5 × platelet concentration)—different protocols (e.g., leukocyte-rich vs. leukocyte-poor PRP, different activation methods) might yield different results; (3) the lack of formal assessment of adverse events beyond immediate procedural complications; and (4) the absence of validated MCID thresholds for some outcome measures (MRC, BMRC, SCV, MCV), which limits the clinical interpretability of those findings.

Based on the findings and limitations of this study, we suggest the following areas for future investigation: (1) prospective, randomized, double-blind, placebo-controlled trials with larger sample sizes to confirm the efficacy of perineural PRP injections; (2) studies comparing different PRP formulations (e.g., leukocyte-rich vs. leukocyte-poor, activated vs. non-activated) and different injection volumes or frequencies (e.g., single vs. multiple injections); (3) trials with longer follow-up periods (≥12 months) to assess the durability of functional gains and to monitor for late complications such as neuroma formation or fibrosis; (4) investigations to identify patient subgroups that are most likely to benefit from PRP (e.g., based on injury grade, time from injury, or baseline serum neurotrophic levels); (5) studies directly comparing perineural (extraneural) vs. intraneural PRP injection techniques to define the optimal risk–benefit balance; and (6) cost-effectiveness analyses to determine whether the modest additional benefits of PRP justify its cost in routine clinical practice.

## 5 Conclusion

In this retrospective observational study, ultrasound-guided perineural injection of leukocyte-poor PRP as an adjunct to conventional rehabilitation was associated with statistically significant improvements in nerve structural remodeling (increased maximum transverse diameter and increased cross-sectional area), neurophysiological parameters (SCV, MCV), and early functional scores (DASH, MRC, BMRC) compared to rehabilitation alone in patients with forearm peripheral nerve injuries. However, the incremental benefit in DASH scores did not reach the minimal clinically important difference, suggesting that the added value of PRP may be modest. Both groups achieved substantial functional recovery from baseline, confirming that structured rehabilitation remains the cornerstone of treatment. While PRP appears safe and may offer additional benefits for selected patients, further prospective, randomized, placebo-controlled trials are needed to confirm its efficacy and define optimal patient selection, preparation protocols, and long-term outcomes.

## Data Availability

The raw data supporting the conclusions of this article will be made available by the authors, without undue reservation.
